# Case Report: Metastatic involvement of the acromioclavicular joint in a patient with papillary carcinoma of the ureter

**DOI:** 10.3389/fsurg.2024.1298556

**Published:** 2025-01-08

**Authors:** Cihangir Türemiş, Mustafa Çeltik, Mehmet Erduran

**Affiliations:** ^1^ Cesme Alper Cizgenakat State Hospital, Izmir, Türkiye; ^2^ Kağızman State Hospital, Kars, Türkiye; ^3^ Department of Orthopedics, Dokuz Eylul University, Izmir, Türkiye

**Keywords:** ureteral papillary carcinoma, acromioclavicular (AC) joint, metastases, clavicula, reconstruction method

## Abstract

Ureteral papillary carcinoma is a rare subtype of urothelial carcinoma, ranking fourth among cancers following prostate (or breast) cancer, lung cancer, and colorectal cancer. Although previous studies have documented bone metastases mainly in the pelvis, spine, ribs, and femur, this case report presents the first recorded instance of metastasis occurring in the acromioclavicular joint. A 62-year-old woman with a history of left flank pain and macroscopic hematuria underwent a left nephroureterectomy, which revealed ureteral papillary carcinoma. Three years later, she reported left shoulder pain, leading to the discovery of a metastatic lesion in the distal clavicle. Approximately 9 cm of metastatic bone was resected while preserving nearby nerve and vascular structures. The resulting bone defect was grafted with a 9-cm bone graft removed from the middle third of the fibula. This case report underscores the importance of considering distant metastases, even in atypical locations, in patients with ureteral papillary carcinoma and aims to share the entire treatment journey and insights gained.

## Introduction

Ureteral papillary carcinoma is a rare form of urothelial carcinoma, ranking fourth among malignancies after prostate (or breast) cancer, lung cancer, and colorectal cancer (1). Upper tract urothelial carcinoma (UTUC) comprises 5%–10% of urothelial cancers, with bladder cancer (BC) being the most common (2). UTUCs peak in those aged 70–90 years and are three times more common in men (3). While most cases remain localized, the metastatic spread can impact multiple organs, mainly via hematogenous and lymphatic pathways, ultimately affecting survival rates (4–6). Bone metastases have been reported in the literature, primarily involving the pelvis, spine, ribs, and femur (7, 8). There are no reported cases of acromioclavicular (AC) involvement in papillary carcinoma of the ureter in the literature, emphasizing the rarity of such metastatic involvement. This case report further broadens the understanding of the course of the disease by showing that the clavicle, acromioclavicular joint, and surrounding tissues may also be involved.

## Case presentation

A 62-year-old woman presented to the urology outpatient clinic with a 6-month history of left flank pain and intermittent macroscopic hematuria. The ultrasound examination revealed significant hydronephrosis along with enlarged renal parenchyma. A computed tomography (CT) scan of the abdomen showed a lesion in the ureter accompanied by proximal dilatation, hydronephrosis, and a dysfunctional ipsilateral renal unit. Retrograde urography revealed several filling defects along with a 2.5-cm lesion featuring a smooth contour, prompting a biopsy. A left nephroureterectomy was performed, and the pathology result was diagnosed as ureteral papillary carcinoma.

**Figure 1:**
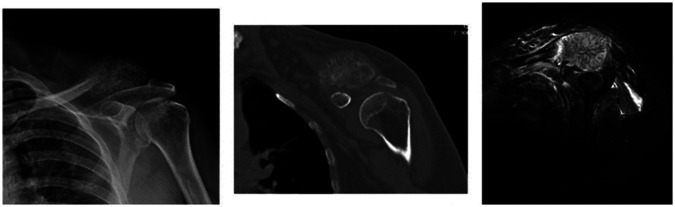
Preoperative images of the patient: direct radiograph, CT, and MRI.

During her postoperative follow-up, 3 years after surgery, the patient reported pain in her left shoulder. Subsequently, an x-ray examination was performed to assess for potential masses, which revealed a heterogeneous solid lesion, measuring up to 6 cm, located at the distal end of the clavicle. The mass was evaluated via magnetic resonance imaging (MRI) and CT scans. The ensuing radiology report indicated the following: “Expansile mass lesion presumed to be metastatic, located in the distal part of the clavicle, accompanied by soft tissue and muscle edema (Figure 1).” The Musculoskeletal Tumor Council assessed the patient and opted for a biopsy. The true-cut biopsy results suggested possible metastases, prompting the need for surgery.

## Surgical management

**Figure 2:**
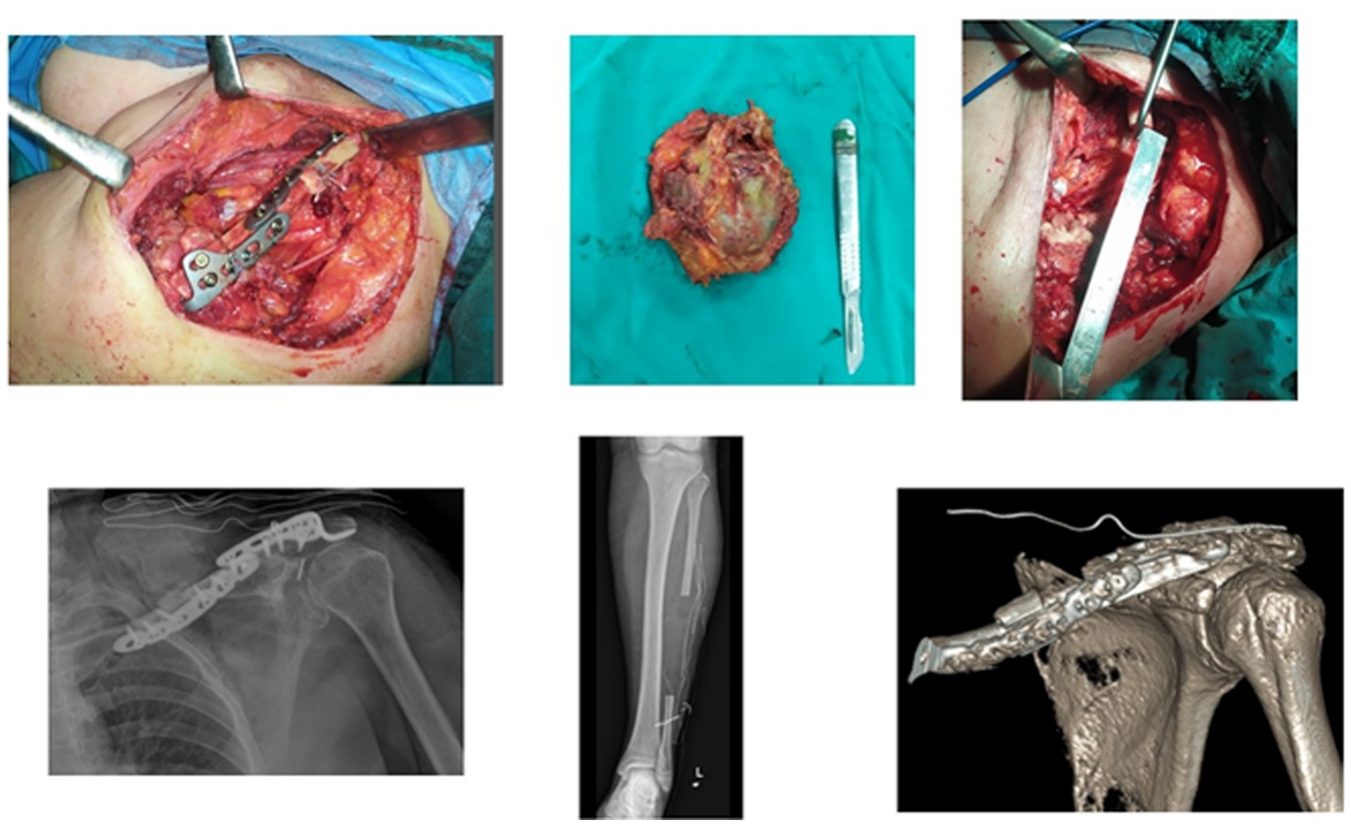
(Top) Images of an intraoperatively removed mass and fixation of a fibular graft endobutton. (Bottom) Perioperative direct radiograph and CT 3D.

After anesthesia preparations, the patient was placed in the supine position. The ipsilateral left leg and left shoulder were prepped and sterile draped. An incision was then made over the left clavicle, allowing for the resection of approximately 9 cm of metastatic bone, while preserving the nerve and vascular structures. The bone defect was subsequently grafted using a 9-cm long bone graft removed from the middle third of the fibula. The removed fibular bone graft was fixed to the medial clavicle with a dynamic compression plate. The distal part of the bone graft was stabilized from the graft to the coracoid using the endobutton method and from the graft to the acromion using a hook plate to ensure both horizontal and vertical stability of the acromioclavicular joint (Figure 2). The patient began isometric shoulder exercises in the second week after the wound site follow-up with a shoulder sling. The material sent to pathology was diagnosed as metastatic ureteral papillary carcinoma. She was referred to the Oncology Department.

## Discussion

This case report highlights the importance of considering distant metastases, even in unusual sites, in patients with ureteral papillary carcinoma. Metastatic recurrence of ureteral papillary carcinoma mainly occurs in the retroperitoneal, pelvic, and supraclavicular lymph nodes, indicating the lymphatic spread of cancer cells (9). The abundant blood supply and broad lymphatic network surrounding the AC joint may promote these metastases. At the same time, cytotoxic chemotherapy agents may damage the lymphatic and vascular structures around the joint, potentially leading to direct invasion. Clinicians may overlook rare metastases and subtle symptoms of patients related to other specialties if they evaluate them solely within their specialty. Therefore, a multidisciplinary approach is necessary to assess diseases involving metastases. Comprehensive multidisciplinary follow-up, examinations, and treatment are vital to prevent early recurrence and further metastasis.

This unusual presentation of metastatic ureteral papillary carcinoma emphasizes the necessity for a comprehensive multidisciplinary evaluation of the patient when metastases are suspected. It also highlights the mechanisms of potential metastasis and their implications for clinical practice and future research.

## Data Availability

The raw data supporting the conclusions of this article will be made available by the authors without undue reservation.
